# GONOME: measuring correlations between GO terms and genomic positions

**DOI:** 10.1186/1471-2105-7-94

**Published:** 2006-02-25

**Authors:** Stefan M Stanley, Timothy L Bailey, John S Mattick

**Affiliations:** 1 Institute for Molecular Bioscience, University of Queensland, Brisbane 4072, Australia

## Abstract

**Background:**

Current methods to find significantly under- and over-represented gene ontology (GO) terms in a set of genes consider the genes as equally probable "balls in a bag", as may be appropriate for transcripts in micro-array data. However, due to the varying length of genes and intergenic regions, that approach is inappropriate for deciding if any GO terms are correlated with a set of genomic positions.

**Results:**

We present an algorithm – GONOME – that can determine which GO terms are significantly associated with a set of genomic positions given a genome annotated with (at least) the starts and ends of genes. We show that certain GO terms may appear to be significantly associated with a set of randomly chosen positions in the human genome if gene lengths are not considered, and that these same terms have been reported as significantly over-represented in a number of recent papers. This apparent over-representation disappears when gene lengths are considered, as GONOME does. For example, we show that, when gene length is taken into account, the term "development" is not significantly enriched in genes associated with human CpG islands, in contradiction to a previous report. We further demonstrate the efficacy of GONOME by showing that occurrences of the proteosome-associated control element (PACE) upstream activating sequence in the *S. cerevisiae *genome associate significantly to appropriate GO terms. An extension of this approach yields a whole-genome motif discovery algorithm that allows identification of many other promoter sequences linked to different types of genes, including a large group of previously unknown motifs significantly associated with the terms 'translation' and 'translational elongation'.

**Conclusion:**

GONOME is an algorithm that correctly extracts over-represented GO terms from a set of genomic positions. By explicitly considering gene size, GONOME avoids a systematic bias toward GO terms linked to large genes. Inappropriate use of existing algorithms that do not take gene size into account has led to erroneous or suspect conclusions. Reciprocally GONOME may be used to identify new features in genomes that are significantly associated with particular categories of genes.

## Background

The Gene Ontology (GO) project [1] arose partly in response to the problem of non-uniform assignment of genomic annotations. Biological databases are notorious for the inconsistency of their annotation terminology, and attempts to apply statistical methods based on annotations across, or even within, genomes face difficulty with this problem. GO addresses this issue by re-expressing annotations using controlled vocabularies, or ontologies; by providing a mechanism for formalizing relationships between qualitative properties (GO terms) that can be associated to genomic features; and by creating a hierarchical structure of these qualities through the use of 'is-a' and 'part-of-a' relationships, allowing one to fit all annotation terms into a "tree" structure (actually a directed acyclic graph) with the most general terms at the root and the most specific terms as leaves. The GO database is broken into three such hierarchies, or aspects: biological process, molecular function and cellular component.

GO has become a popular way of analyzing sets of genes to find under- or over-represented terms associated with that set of genes, especially in expression micro-array datasets. One may, for example, apply a "GO analysis" to sets of up- or down-regulated genes to assess which processes or functions are undergoing coordinated regulation. A variety of web-based tools exist that allow one to enter a list of gene identifications and find the over- and under-represented GO terms associated to those genes – for example GOstat [2] and GO::TermFinder [3].

In this work, we consider the slightly different task of determining whether a *set of genomic positions *is associated with any GO terms. This situation arises in many contexts (see, e.g. [4,5]). One might, for example, wish to determine if a particular regulatory sequence motif is significantly associated with genes involved in any particular pathway.

The typical "GO analysis" begins with a set of genes and uses a random model that assumes that each gene in the genome is equally likely *a priori *to be included in the set. This assumption is inappropriate when the input is a set of genomic positions rather than a set of genes. Due to the varying length of genes and intergenic distances, randomly selected genomic positions are much more likely to fall within large genes or within large adjacent intergenic regions. Therefore, when determining which (if any) GO terms are significantly associated with a set of genomic positions, a different random model is required.

As an illustration, imagine that we are given a set of genomic positions and asked to determine with which GO terms they are associated. Note, firstly, that the GO database maps genes to terms, so we must define how we are going to map genomic positions to genes. A natural way to associate GO terms with genomic positions is to associate each position with a single gene, and, transitively, with that gene's GO terms: *position*→*gene*→*GO Term*. In this example, we map each (strand-specific) genome position falling within a gene to that gene. Suppose then that the genome consists of five 1 Kb genes annotated as metabolic and one 1 Mb gene annotated as meiotic. A randomly chosen genomic sequence in this genome is 10^6^/5000 = 200 times more likely to lie within the meiotic gene than within any of the metabolic genes. Therefore, a random model that assumes all genes (and, hence, their GO terms) are equally likely to be selected is clearly inappropriate. Using such a model would cause randomly chosen genomic positions to (erroneously) correlate with GO terms associated with the meiotic gene. Thus a new approach is required to assess the statistical significance of GO terms associated to genomic positions that explicitly considers gene length, as opposed to the event-based associations currently used with gene expression data.

## Results

### GONOME: Gene Ontology correlations in the genome

We have developed a new application, called GONOME [6], which calculates the statistical significance of the correlation between a set of genomic positions and their associated Gene Ontology terms. GONOME does this by applying a random model that assumes that each *position *(rather than each *gene*) in the portion of the genome under consideration is equally likely. This implies that the chance of a uniformly distributed random position "hitting" (lying within, or adjacent to) a particular genomic region is proportional to the size of the region, removing the bias toward large genes caused by considering all genes equally probable.

GONOME takes as input a set of genomic positions and a genome annotated with the locations of genes. The positions in the input set may be strand-specific or not. If a DNA strand is not specified, GONOME replaces the position with two positions: one on each DNA strand.

GONOME also allows the user to define which genomic regions are of interest in a particular analysis. The upstream, transcribed and downstream regions of each gene may be associated with its GO terms or treated as 'unscored'. These regions are linked to the GO terms associated with the gene's GO terms (If a gene has no associated GO terms, its regions are associated with the "placeholder" term "NO_GO"). When the upstream and downstream regions of two adjacent genes overlap, GONOME treats the positions in the overlap as lying in both regions. All positions not lying in the upstream, transcribed or downstream region of any gene are also considered unscored, have no GO terms associated with them, and the user can choose to either include or exclude them from the analysis.

The user can also control the allowed size of the associated upstream and downstream regions via configurable "cutoffs". This is a useful feature because which genomic positions are of interest depends on the organism being studied as well as the type of positions being analyzed. For example, a position 100 bp upstream of a gene might naturally be associated with that gene, a position 500 bp upstream of one gene and 500 bp downstream of another might be ascribed to either or both of the flanking genes, while one 10 Kbp upstream and downstream from the nearest genes might not be associated at all. Such judgments are partly dependent on the size of the genome and the intergenic regions (e.g. yeast vs. human). Additionally, if the positions in the input set are (putative) promoters, one might only be interested in the region upstream of the gene, and only if within 500 bp.

Finally, rather than merely counting the number of times each GO term is associated with a genomic position in the input set, GONOME allows the user to specify weights for each type of region: upstream, transcribed and downstream. This allows the user to tailor a "correlation scoring function" appropriate to the biological questions being asked.

### Over-represented terms from random positions

**Figure 1:**
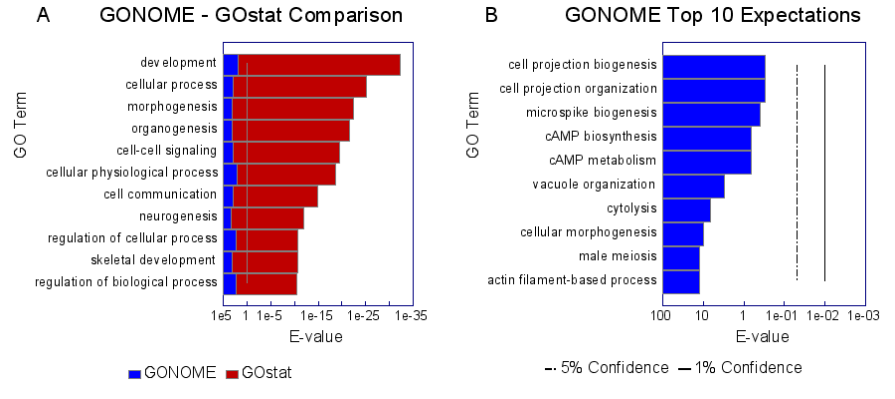
**GONOME and GOstat output on random positions**. Panel **A **compares GONOME and GOstat analyses of 30,000 randomly selected positions in the human genome. The *E*-values of the top 10 over-represented GO Terms as found by GOstat (red), and the values GONOME derives for the same terms (blue). Panel **B **shows the top ten over-represented terms according to GONOME. *E*-values were calculated as described in Methods.

To validate the approach and compare it with previous approaches, we compared the statistical significance of the GO associations of a set of 30,000 randomly chosen strand-specific positions in the human genome calculated by GONOME and GOstat [2]. The genomic positions were generated uniformly across both strands of the genome. We set the GONOME parameters so that all three types of region (upstream, downstream and the transcribed gene, including its introns and UTRs) were included in the analysis and that all intergenic regions were ascribed as the downstream and upstream regions of the respective adjacent genes. For input to GOstat, a set of genes corresponding to the randomly chosen set of genomic positions was constructed similarly by adding the gene for positions falling within the boundaries of the transcribed gene, and by adding both the upstream and downstream genes for positions falling within intergenic sequences. The results are shown in Figure 1.

It is apparent from Figure 1A that GOstat reports numerous GO terms as being over-represented among the random positions. The most over-represented term is 'development', with the estimated probability of random occurrence (*E*-value) being 2.41 × 10^-31 ^– a number that would be hard to consider insignificant. This result may be understood by observing that many of the over-represented terms are associated with genes encoding developmental regulators and/or membrane proteins, genes longer than most – the average length of all GO annotated genes is 106.5 Kb (5257 genes) (note that non-GO annotated genes have an average length of 34.4 Kb, suggesting that these may not include distal exons and/or include artifacts), whereas the average of those annotated as development is 133.9 Kb (770 genes), cellular process 125.8 Kb (2832 genes), morphogenesis 152.2 Kb (466 genes), and organogenesis 158.3 Kb (392 genes). In contrast, GONOME finds no terms occurring significantly more often than expected at random (Figure 1A). What is more, the top ten GONOME *E*-values range from values of about one to about ten, as they should when the genomic positions are chosen at random. This result clearly demonstrates the inappropriateness of considering all genes as equally likely when analyzing genomic positions. This error has been made in a number of recent papers, which typically ascribe development as a significantly enriched term. For example, more than half of the over-represented terms reported in Table 1 from Seipel *et al*. [5] are also predicted as over-represented by GOstat applied to sets of random positions. Another example is provided by the Robinson *et al*. [7] analysis of CpG islands in the human genome, which we re-analyze in the next section. On the other hand, the general gene categories (RNA processing, regulation of transcription and development) reported as being significantly associated with ultraconserved elements [4] remain significantly associated using GONOME (data not shown).

**Table 1:** Novel putative motifs found by GONOME. GONOME was used to find over-represented GO terms associated with each possible n-mer (for *n *from 5 to 11) in the *S. Cerevisiae *genome, here are some of the significant motifs not reported elsewhere. (Motifs are expressed in IUPAC extended DNA alphabet: K is G or T; V is G, C or A; B is G or T or C; S is G or C; M is A or C)

**Motif**	**GO Term**	**GONOME *E*-value**	**Transcription Factors**
CCCCTAAAA	vitamin metabolism	2.5e-7	ADR1, NRG1
GCCCTAA	rRNA modification	1.1e-5	NRG1
TCCGCGG	Response to drug	8.7e-11	SUT1, STB5
GGVBCCSG	Translation	3.3e-30	-
CACGTGA	Sulfur amino acid metabolism	6.5e-10	CBF1
GKKGSMAAA	Protein catabolism	1.0e-10	
TGGCAAA	Protein catabolism	7.0e-4	-

### Human CpG Islands

**Figure 2:**
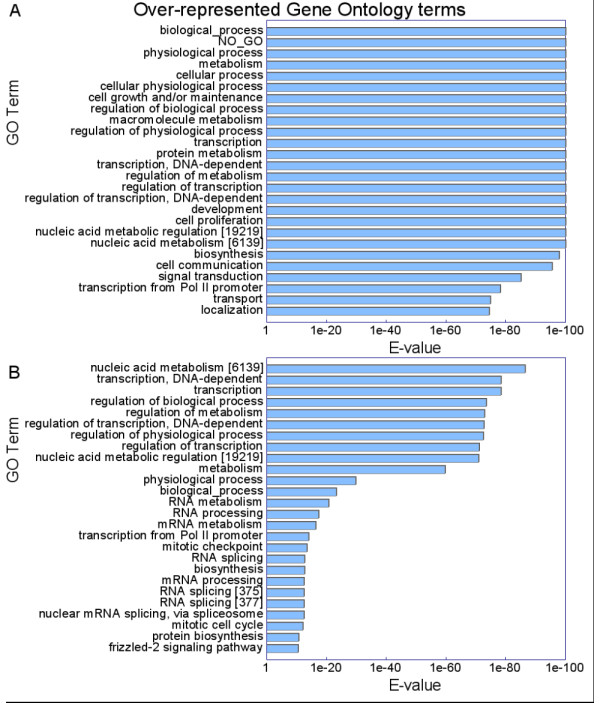
**Over-represented gene ontology terms associated with human CpG islands**. Over-represented gene ontology terms associated with human CpG islands as determined by GONOME when (**A**) unscored regions are included in the analysis and when (**B**) unscored regions are excluded from the analysis. The *E*-values of the 25 most over-represented GO process associated with CpG islands in the human genome in each case are shown. The image is the actual output of the GONOME application, save that long GO terms have been replaced with shorter equivalents and their GO identification numbers provided in brackets.

A CpG island is a cluster of CG dinucleotides, which appear as obvious features in mammalian genomes wherein only a quarter of the expected number of CG dinucleotides occur. CpG islands have previously been shown to be strongly associated with 'housekeeping' genes [8-12], but were recently reported to be also significantly associated with genes annotated with the GO term "development" using a chi-squared based method [7]. Following Robinson *et al*. [7], we applied GONOME to the positions of CpG islands in the human genome, restricting the scored positions to those occurring within genes and their 'promoter' regions (2000 bp upstream), against a null model that compares the observed pattern to that expected from random positions occurring uniformly throughout the entire genome (i.e. including the regions outside genes and their immediate 5'-promoter regions, termed the 'unscored' regions) (Figure 2).

The results show that, using the null model of the random incidence of CpG islands in the entire genome, the GO root node 'biological_process' and other broad functional descriptors are very significantly over-represented terms associated with CpG islands (Figure 2A). This occurs because CpG islands occur more often in or near the beginnings of genes *per se *than would be expected for a uniform distribution of random positions across the genome. Other generic terms indicating housekeeping functions as well as the term 'NO_GO' (the placeholder for genes without associated GO annotations) also receive significant *E*-values. Thus, if GONOME is applied against the whole genome in this way, and general GO terms are reported as being significantly over-represented, one may conclude that the chosen feature (in this case CpG islands) is strongly associated with genes and their promoters, genome-wide. However, while this may be generally informative, the strength of the signal obscures potential specific associations of the feature in question with particular subsets of genes, and thus one needs to be judicious about the choice of the null model.

The alternative null model excludes unscored positions in the genome, thereby calculating the chance that any GO term attains its score given the actual number of scoring positions. Using this model one can better assess whether there is a significant bias in the association of CpG islands with specific GO terms. When we repeated the analysis with this model, we found that GO terms with a "housekeeping" nature, such as those including the words 'metabolism,' 'transcription' and 'regulation' predominate (Figure 2B). This confirms the strong association of CpG islands with housekeeping genes, as well as the over-representation of the most significantly enriched term found in CpG island associated genes from Table 2 in Robinson *et al*. [7], 'regulation of transcription, DNA dependent'. However, of the remaining nine biological process terms in that table, seven are found to be over-represented in four runs of 30,000 *random *positions through GOstat. It should be borne in mind, however, that the previous analysis also used a tissue-specific metric on gene expression (the distribution of ESTs belonging to the same UniGene cluster) [7] that ours does not.

The dataset used in Figure 2 has been masked for repetitive sequences, and contains 67,697 positions. However, when GONOME is used with the same parameters on the unmasked set of 102,064 positions, virtually identical results are found. This demonstrates that GONOME is robust to noise – the repetitive positions showed no significantly over-represented terms (graphs available on the website). Full graphs and corresponding results for the murine and yeast genomes, as well as correlations with terms in the function and component GO hierarchies are available online [13].

### Whole-genome motif discovery with GONOME

**Figure 3:**
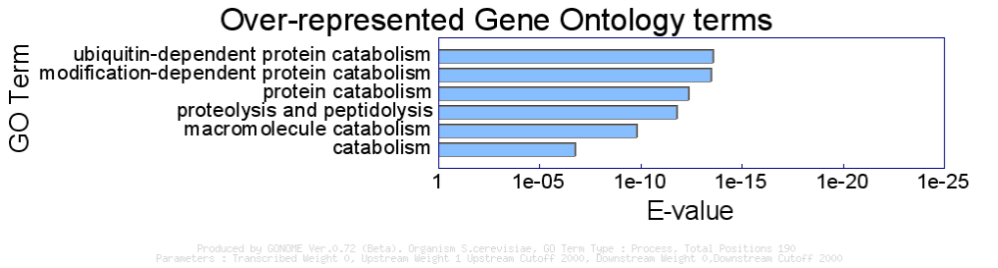
**GONOME analysis of PACE elements**. Figure legend text. Over-represented GO terms associated with positions in the *S. cerevisiae *genome of the proteosome associated control element (PACE) upstream activating sequence (UAS), 5'-GGTGGCAAA-3'. "Locus" refers in general to a gene and its associated upstream and downstream regions, which are "Hit" once when a position falls within any associated region.

In another test of GONOME, we used the positions of all 190 exact occurrences of the proteosome associated control element (PACE) upstream activating sequence (UAS), 5'-GGTGGCAAA-3' [14] in the *S. cerevisiae *genome. The PACE sequence motif is known to be present in the upstream regions of 27 proteasomal genes, and a number of ubiquitin-proteasomal genes. As we are interested in a UAS, we directed GONOME to include only (2000 bp) upstream regions in the analysis, against a null model of the whole genome (the results of which barely differ from null model that only considers the scored regions, because highly gene-specific terms do not give a broader signal associated with genes generally). As expected, GONOME reveals significant over-representation of terms for the appropriate (proteasomal) processes (Figure 3) indicating its accuracy.

This analysis naturally raises the question of whether GONOME could be applied on a genome-wide scale to extract functionally-linked but as yet unrecognized UAS sequences that may bind other transcription factors. To this end, we used GONOME to analyze the positions in the yeast genome of all possible *k*-mers (for *k *in the range 5 to 11) looking for any that showed significant over-representation with particular GO terms. The complete results are available at [13], and largely recapitulate results of a similar study carried by Cora et al [15], without requiring the prior isolation of upstream sequences associated to groups of genes. Moreover, GONOME automatically identified several additional motifs, including the PACE element, not reported in Cora *et al*. (Table 1).

One interesting finding is that it would appear that the PACE element (GGTGGCAAA) is actually a specific subset of a more general motif (GKKGSMAAA) associated with most protein catabolism genes. Whereas the PACE element is found in the 2000 bp upstream of 27 genes annotated as involved in protein metabolism, with 18 of those also being annotated with 'protein catabolism', the related motif TGGCAAA (underlined above, a subset of both the PACE and the general motif) occurs in the upstream regions of 56 protein catabolism genes, and of 65 genes involved in 'macromolecule catabolism', perhaps implying greater promiscuity for the PACE associated transcription factor, *RPN4 *[14,16,17], than is currently accepted, or the existence of another transcription factor with overlapping specificity.

Another noteworthy finding is that, along with the previously reported large groups of motifs associated with ribosome biogenesis and DNA replication, GONOME identified a large group of previously unknown motifs significantly associated with the terms 'translation' and 'translational elongation'. A set of genes is known to be regulated as a group, for example, in stress induced inhibition of translation [18]. The full set of over-represented motifs found in this analysis can be also found in the resources section of the website.

## Discussion

GONOME provides a flexible way to examine correlations between GO terms or other annotated features of genes and sets of genomic positions. Configurable parameters allow the genomic areas of interest to be defined and given relative weights. Two different null models allow the user to consider or exclude non-associated regions of the genome from the statistical analysis. GONOME reports correlations as *E*-values, conservatively accounting for the testing of multiple hypotheses. The statistical model accurately accounts for the varying length of genomic regions.

GONOME is also a useful adjunct to many positional genomic analysis methods (*e.g*., BLAST [19] or linkage analysis) providing a generally applicable method for enhancing understanding of the biological significance of any set of genomic positions. It should also be noted that the methodology is not restricted to GO term data. In theory, any type of annotation data that can be associated with genes can be accommodated.

As with any statistical method, some caution needs to be used in the choice of parameters in order to achieve the best results. We have observed that, in large genomes, limiting the upstream and downstream cutoff distances to around 10 Kbp avoids penalizing genes next to gene deserts. It should also be borne in mind that larger genes may and probably do have more extended regions of regulatory information, including that in introns, and therefore it may be appropriate, depending on the context of the question, to (also) use incidence-based statistical packages such as GOStat to obtain another perspective on regulatory correlations.

Another issue can arise when multiple "hits" to a small number of genes occurs due to clustering. For example, if considering the terms associated to transposon positions, a heavily invaded gene with 100 Alu hits causes all terms associated with that gene to have significant *E*-values. While this is indeed a statistically significant result, it may obscure a more interesting genome-wide pattern. These types of issues can be handled by representing clusters of nearby positions with a single representative position. The GONOME software package includes a simple clustering routine for this purpose. The clustering routine finds all chains of positions separated by less than a (user-specified) threshold distance, and replaces them with the midpoint of the chain.

GONOME presently reports *E*-values computed by applying the conservative Bonferroni adjustment to *p*-values to correct for multiple hypotheses. In the future this might be extended to methods such as False Discovery Rate [20-22]. However, while the optimal way to account for multiple hypotheses in the densely inter-related gene ontology hierarchy remains an open question, the Bonferroni approach seems prudent.

## Conclusion

GONOME provides a method for assessing the statistical significance of the association of genomic features with particular types of genes, and enables the correction of artifacts associated with variable gene size when using event-based statistical packages. GONOME may be tailored to specify the length of flanking sequences included in the analysis as well as used as a tool to discover new sequence motifs that are significantly associated with particular types of genes.

## Methods

### Correlations between GO terms and genomic positions

The objective of GONOME is to ascertain if a set of genomic positions is correlated with some biological property ("term") as annotated in the gene ontology (GO) database, and to compute the probability of such a correlation occurring at random. The GO database associates terms with genes, not genomic positions, so one first must define when a genomic position is considered to be associated with a GO term. A scoring function is then defined that measures the degree of correlation between a given go term, *T*, and a set of genomic positions, **X **= {*x*_1_,*x*_2_,...,*x*_*n*_}. Finally, to determine if the degree of correlation is statistically significant, the random distribution of the scoring function is computed.

### Associating GO terms with genomic positions

Each genomic position, *x*_*i*_, in the input set, **X **= {*x*_1_,*x*_2_,...,*x*_*n*_}, consists of a chromosome or contig identifier, a position on that chromosome or contig, and a strand (*i.e*., Watson or Crick). (The user may specify unstranded positions in the input to GONOME, but these are each replaced by two positions, one on each DNA strand.) GONOME associates all the GO terms associated with a gene with each genomic position (on the gene's DNA strand) that falls within the upstream, transcribed or downstream region of the gene. GONOME permits the user to define the extents (in base pairs) of upstream and downstream regions. The downstream region of a gene extends until the start of the flanking gene's transcribed region or until the downstream cutoff is reached, whichever comes first. A similar definition applies to upstream regions. If the downstream region of a gene overlaps the upstream region of the flanking gene, the shared genomic positions (herein called an "overlap region") are associated with the GO terms of both genes. Genomic positions that are not part of any gene's upstream, downstream or transcribed regions (as defined above), are treated as unscored by GONOME and are not associated with any GO terms.

### The correlation scoring function

GONOME assigns a "weight" to each type of genomic region that reflects the "strength" of the association between positions in the given type of region and the GO terms of the corresponding gene. The weights for the upstream, downstream, transcribed and overlap regions are called *w*_*u*_, *w*_*d*_, *w*_*t *_and *w*_*o*_, respectively. An upstream, transcribed or downstream region is defined to be "annotated with GO term *T*" if its associated gene is. An overlap region is defined to be annotated with *T *if *both *flanking genes are. Using these definitions, GONOME's score for the association between a single genomic position, *x*, and a GO term, *T*, in terms of the region weights is

s(x,T)={wuif x is in an upstream region annotated with T,wtif x is in a transcribed region annotated with T,wdif x is in a downstream region annotated with T,wo=wu+wdif x is in an overlap region annotated with T,0otherwise.
 MathType@MTEF@5@5@+=feaafiart1ev1aaatCvAUfKttLearuWrP9MDH5MBPbIqV92AaeXatLxBI9gBaebbnrfifHhDYfgasaacH8akY=wiFfYdH8Gipec8Eeeu0xXdbba9frFj0=OqFfea0dXdd9vqai=hGuQ8kuc9pgc9s8qqaq=dirpe0xb9q8qiLsFr0=vr0=vr0dc8meaabaqaciaacaGaaeqabaqabeGadaaakeaacqWGZbWCcqGGOaakcqWG4baEcqGGSaalcqWGubavcqGGPaqkcqGH9aqpdaGabaqaauaabaqafiaaaaqaaiabdEha3naaBaaaleaacqWG1bqDaeqaaaGcbaGaeeyAaKMaeeOzayMaeeiiaaIaemiEaGNaeeiiaaIaeeyAaKMaee4CamNaeeiiaaIaeeyAaKMaeeOBa4MaeeiiaaIaeeyyaeMaeeOBa4MaeeiiaaIaemyDauNaemiCaaNaem4CamNaemiDaqNaemOCaiNaemyzauMaemyyaeMaemyBa0MaeeiiaaIaeeOCaiNaeeyzauMaee4zaCMaeeyAaKMaee4Ba8MaeeOBa4MaeeiiaaIaeeyyaeMaeeOBa4MaeeOBa4Maee4Ba8MaeeiDaqNaeeyyaeMaeeiDaqNaeeyzauMaeeizaqMaeeiiaaIaee4DaCNaeeyAaKMaeeiDaqNaeeiAaGMaeeiiaaIaemivaqLaeiilaWcabaGaem4DaC3aaSbaaSqaaiabdsha0bqabaaakeaacqqGPbqAcqqGMbGzcqqGGaaicqWG4baEcqqGGaaicqqGPbqAcqqGZbWCcqqGGaaicqqGPbqAcqqGUbGBcqqGGaaicqqGHbqycqqGGaaicqWG0baDcqWGYbGCcqWGHbqycqWGUbGBcqWGZbWCcqWGJbWycqWGYbGCcqWGPbqAcqWGIbGycqWGLbqzcqWGKbazcqqGGaaicqqGYbGCcqqGLbqzcqqGNbWzcqqGPbqAcqqGVbWBcqqGUbGBcqqGGaaicqqGHbqycqqGUbGBcqqGUbGBcqqGVbWBcqqG0baDcqqGHbqycqqG0baDcqqGLbqzcqqGKbazcqqGGaaicqqG3bWDcqqGPbqAcqqG0baDcqqGObaAcqqGGaaicqWGubavcqGGSaalaeaacqWG3bWDdaWgaaWcbaGaemizaqgabeaaaOqaaiabbMgaPjabbAgaMjabbccaGiabdIha4jabbccaGiabbMgaPjabbohaZjabbccaGiabbMgaPjabb6gaUjabbccaGiabbggaHjabbccaGiabdsgaKjabd+gaVjabdEha3jabd6gaUjabdohaZjabdsha0jabdkhaYjabdwgaLjabdggaHjabd2gaTjabbccaGiabbkhaYjabbwgaLjabbEgaNjabbMgaPjabb+gaVjabb6gaUjabbccaGiabbggaHjabb6gaUjabb6gaUjabb+gaVjabbsha0jabbggaHjabbsha0jabbwgaLjabbsgaKjabbccaGiabbEha3jabbMgaPjabbsha0jabbIgaOjabbccaGiabdsfaujabcYcaSaqaaiabdEha3naaBaaaleaacqWGVbWBaeqaaOGaeyypa0Jaem4DaC3aaSbaaSqaaiabdwha1bqabaGccqGHRaWkcqWG3bWDdaWgaaWcbaGaemizaqgabeaaaOqaaiabbMgaPjabbAgaMjabbccaGiabdIha4jabbccaGiabbMgaPjabbohaZjabbccaGiabbMgaPjabb6gaUjabbccaGiabbggaHjabb6gaUjabbccaGiabd+gaVjabdAha2jabdwgaLjabdkhaYjabdYgaSjabdggaHjabdchaWjabbccaGiabbkhaYjabbwgaLjabbEgaNjabbMgaPjabb+gaVjabb6gaUjabbccaGiabbggaHjabb6gaUjabb6gaUjabb+gaVjabbsha0jabbggaHjabbsha0jabbwgaLjabbsgaKjabbccaGiabbEha3jabbMgaPjabbsha0jabbIgaOjabbccaGiabdsfaujabcYcaSaqaaiabicdaWaqaaiabb+gaVjabbsha0jabbIgaOjabbwgaLjabbkhaYjabbEha3jabbMgaPjabbohaZjabbwgaLjabb6caUaaaaiaawUhaaaaa@44B5@

GONOME's scoring function for the association between the input set, **X**, and GO term, *T*, is the sum of the association scores of each genomic position in **X**,

S(X,T)=∑x∈Xs(x,T).
 MathType@MTEF@5@5@+=feaafiart1ev1aaatCvAUfKttLearuWrP9MDH5MBPbIqV92AaeXatLxBI9gBaebbnrfifHhDYfgasaacH8akY=wiFfYdH8Gipec8Eeeu0xXdbba9frFj0=OqFfea0dXdd9vqai=hGuQ8kuc9pgc9s8qqaq=dirpe0xb9q8qiLsFr0=vr0=vr0dc8meaabaqaciaacaGaaeqabaqabeGadaaakeaacqWGtbWucqGGOaakieqacqWFybawcqGGSaalcqWGubavcqGGPaqkcqGH9aqpdaaeqbqaaiabdohaZjabcIcaOiabdIha4jabcYcaSiabdsfaujabcMcaPiabc6caUaWcbaGaemiEaGNaeyicI4Sae8hwaGfabeqdcqGHris5aaaa@41C7@

**Figure 4:**
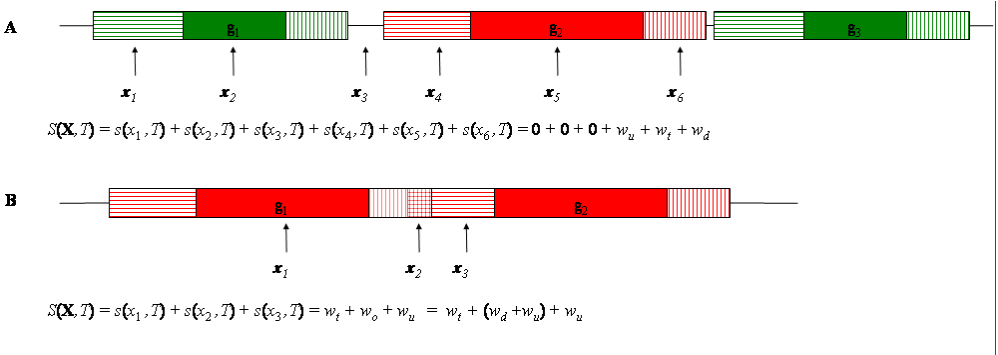
**GONOME scoring function: *S*(X, *T*)**. Genomic positions in the input set X are shown with arrows. Regions belonging to genes annotated with GO term *T *are shaded red. Regions belonging to other genes are shaded green. Transcribed regions are shown in solid color. Upstream (downstream) regions are shown with horizontal (vertical) crosshatching. Unscored regions are shown as horizontal black lines. Positions in green and unscored regions receive association score of zero. Other positions receive association scores equal to the appropriate region weight. Panel **A **illustrates the simplest case where upstream and downstream regions of adjacent genes do not overlap. In panel **B**, the position *x*_2 _lies in the "overlap" region of two "red" genes, so its score is the sum of the upstream and downstream weights.

The GONOME correlation scoring function is illustrated in Figure 4.

The user may choose the value of each region weight in order to design a correlation scoring function appropriate to the task at hand. Weights must be non-negative. For example, to have the scoring function simply count the number of positions in the input set that lie within or near genes associated with GO term *T*, the GONOME user can set the region weights to *w*_*u *_= *w*_*t *_= *w*_*d *_= 1. (This is not strictly true if any input positions lie in the overlap regions of two "red" genes as shown in Figure 4B). Such positions are effectively counted twice. On the other hand, if the user believes that positions in transcribed regions should be stronger evidence of association with term *T*, this intuition can be incorporated into the scoring function by setting the weights to, for example, *w*_*u *_= *w*_*d *_= 1/2 and *w*_*t *_= 1. As a final, simpler, example, setting the weights to *w*_*u *_= 1 and *w*_*u *_= *w*_*d *_= 0 will cause the scoring function to count only positions within upstream regions, as might be appropriate when studying promoter regions.

### Determining statistical significance

Sufficiently large values of the scoring function *S*(**X**,*T*) indicate that genomic positions annotated with GO term *T *are overrepresented in the set **X**. To determine how large is "large enough," the probability that *S*(**X**,*T*) ≥ *S *is estimated under the null assumption that the set of positions in **X **are chosen randomly from some "universe" of positions. This probability is commonly referred to as the *p*-value of *S*. To adjust for testing multiple hypotheses, we convert *p*-values to *E*-values by multiplying by the number of terms in the GO ontology. This is also referred to as the "Bonferroni adjustment" [23] and gives a conservative estimate of the expected number of GO terms that would score *S *or more when the set of positions, **X**, is uncorrelated with any GO term.

The "universe" of positions referred to in the previous paragraph usually will be just the "genic" positions – the upstream, downstream, transcribed and overlap regions of genes. Alternatively, the user can specify that the universe include all positions in the genome.

GONOME computes score *p*-values by summing the (approximate) probabilities of all possible ways to achieve a score of *S *or greater. Each possible score is the sum of a total of *n *weights, one for each element in **X**. So, if variables *u*, *t*, *d *and *o *represent the number of positions in **X **that lie in upstream, transcribed, downstream and overlap regions, respectively, the correlation score is *S*(**X**,*T*) = *uw*_*u *_+ *tw*_*t *_+ *dw*_*d *_+ *ow*_*o*_. Let Pr(*n*,*u*,*t*,*d*,*o*) be the probability that, out of *n *randomly chosen genomic positions, *u*, *t*, *d *and *o*, respectively are in upstream, transcribed, downstream and overlap regions. Then, by definition, the *p*-value of score *S *is

Pr⁡(S(X,T)≥S)=∑u=0n∑t=0n−u∑d=0n−u−t∑o=⌈(S−uwu−twt−dwd)/wo⌉n−u−t−dPr⁡(n,u,t,d,o),
 MathType@MTEF@5@5@+=feaafiart1ev1aaatCvAUfKttLearuWrP9MDH5MBPbIqV92AaeXatLxBI9gBaebbnrfifHhDYfgasaacH8akY=wiFfYdH8Gipec8Eeeu0xXdbba9frFj0=OqFfea0dXdd9vqai=hGuQ8kuc9pgc9s8qqaq=dirpe0xb9q8qiLsFr0=vr0=vr0dc8meaabaqaciaacaGaaeqabaqabeGadaaakeaacyGGqbaucqGGYbGCcqGGOaakcqWGtbWucqGGOaakieqacqWFybawcqGGSaalcqWGubavcqGGPaqkcqGHLjYScqWGtbWucqGGPaqkcqGH9aqpdaaeWbqaamaaqahabaWaaabCaeaadaaeWbqaaiGbccfaqjabckhaYjabcIcaOiabd6gaUjabcYcaSiabdwha1jabcYcaSiabdsha0jabcYcaSiabdsgaKjabcYcaSiabd+gaVjabcMcaPaWcbaGaem4Ba8Maeyypa0ZaaCWaaeaacqGGOaakcqWGtbWucqGHsislcqWG1bqDcqWG3bWDdaWgaaadbaGaemyDauhabeaaliabgkHiTiabdsha0jabdEha3naaBaaameaacqWG0baDaeqaaSGaeyOeI0IaemizaqMaem4DaC3aaSbaaWqaaiabdsgaKbqabaWccqGGPaqkcqGGVaWlcqWG3bWDdaWgaaadbaGaem4Ba8gabeaaaSGaayP74laawMp+aaqaaiabd6gaUjabgkHiTiabdwha1jabgkHiTiabdsha0jabgkHiTiabdsgaKbqdcqGHris5aaWcbaGaemizaqMaeyypa0JaeGimaadabaGaemOBa4MaeyOeI0IaemyDauNaeyOeI0IaemiDaqhaniabggHiLdaaleaacqWG0baDcqGH9aqpcqaIWaamaeaacqWGUbGBcqGHsislcqWG1bqDa0GaeyyeIuoaaSqaaiabdwha1jabg2da9iabicdaWaqaaiabd6gaUbqdcqGHris5aOGaeiilaWcaaa@9037@

#### Equation 1

provided that *w*_*o *_≠ 0. (If any region weights are zero, those regions are treated as unscored regions, and Equation 1 is modified to only sum over regions with non-zero weights.)

GONOME estimates Pr(*n*,*u*,*t*,*d*,*o*) in Equation 1 using the multinomial distribution

M(u,t,d,o,z,pu,pt,pd,po,pz)=n!∏r∈{u,t,d,o,z}r!∏r∈{u,t,d,o,z}(pr)r,
 MathType@MTEF@5@5@+=feaafiart1ev1aaatCvAUfKttLearuWrP9MDH5MBPbIqV92AaeXatLxBI9gBaebbnrfifHhDYfgasaacH8akY=wiFfYdH8Gipec8Eeeu0xXdbba9frFj0=OqFfea0dXdd9vqai=hGuQ8kuc9pgc9s8qqaq=dirpe0xb9q8qiLsFr0=vr0=vr0dc8meaabaqaciaacaGaaeqabaqabeGadaaakeaacqWGnbqtcqGGOaakcqWG1bqDcqGGSaalcqWG0baDcqGGSaalcqWGKbazcqGGSaalcqWGVbWBcqGGSaalcqWG6bGEcqGGSaalcqWGWbaCdaWgaaWcbaGaemyDauhabeaakiabcYcaSiabdchaWnaaBaaaleaacqWG0baDaeqaaOGaeiilaWIaemiCaa3aaSbaaSqaaiabdsgaKbqabaGccqGGSaalcqWGWbaCdaWgaaWcbaGaem4Ba8gabeaakiabcYcaSiabdchaWnaaBaaaleaacqWG6bGEaeqaaOGaeiykaKIaeyypa0ZaaSaaaeaacqWGUbGBcqGGHaqiaeaadaqeqbqaaiabdkhaYjabcgcaHaWcbaGaemOCaiNaeyicI4Saei4EaSNaemyDauNaeiilaWIaemiDaqNaeiilaWIaemizaqMaeiilaWIaem4Ba8MaeiilaWIaemOEaONaeiyFa0habeqdcqGHpis1aaaakmaarafabaGaeiikaGIaemiCaa3aaSbaaSqaaiabdkhaYbqabaGccqGGPaqkdaahaaWcbeqaaiabdkhaYbaaaeaacqWGYbGCcqGHiiIZcqGG7bWEcqWG1bqDcqGGSaalcqWG0baDcqGGSaalcqWGKbazcqGGSaalcqWGVbWBcqGGSaalcqWG6bGEcqGG9bqFaeqaniabg+GivdGccqGGSaalaaa@7FA3@

#### Equation 2

where *z *= *n *- *u *- *t *- *d *- *o *is the number of positions in **X **that lie in unscored regions, and *p*_*r *_is the fraction of positions of type *r *in the "universe" of positions. Equation 2 will be a good estimate of Pr(*n*,*u*,*t*,*d*,*o*) as long as *n *is small relative to the size of the "universe," so that the probability of randomly choosing the same position twice as very small [24]. This is because Equation 2 represents the probability of randomly selecting the specified numbers (*u*, *t*, *d*, *o *and *z*) of different types of positions in *n *random selections *with replacement*, whereas the correct analogy should be selecting *without replacement*, since the input (**X**) is a set and a single position cannot appear (*i.e*., be selected) more than once.

### Optimizing the *p*-value computation

When all region weights are non-zero, computing Equation 1 requires O(*n*^4^) operations. This becomes prohibitive for large values of *n*. GONOME reduces the computational requirements by a factor of *n *by truncating the innermost summation when the variable *o *is sufficiently large that Pr(*n*,*u*,*t*,*d*,*o*) is negligible. GONOME truncates the sum over *o *when Pr(*n*,*u*,*t*,*d*,*o*) is decreasing and Pr⁡(n,u,t,d,o)<εn2p
 MathType@MTEF@5@5@+=feaafiart1ev1aaatCvAUfKttLearuWrP9MDH5MBPbIqV92AaeXatLxBI9gBaebbnrfifHhDYfgasaacH8akY=wiFfYdH8Gipec8Eeeu0xXdbba9frFj0=OqFfea0dXdd9vqai=hGuQ8kuc9pgc9s8qqaq=dirpe0xb9q8qiLsFr0=vr0=vr0dc8meaabaqaciaacaGaaeqabaqabeGadaaakeaacyGGqbaucqGGYbGCcqGGOaakcqWGUbGBcqGGSaalcqWG1bqDcqGGSaalcqWG0baDcqGGSaalcqWGKbazcqGGSaalcqWGVbWBcqGGPaqkcqGH8aapdaWcaaqaaGGaciab=v7aLbqaaiabd6gaUnaaCaaaleqabaGaeGOmaidaaaaakiabdchaWbaa@422E@, where *p *is the current value of the sum (Equation 1) and ε is a user-selected error threshold. It can be shown that Pr(*n*,*u*,*t*,*d*,*o*) decreases monotonically in *o *once it reaches its maximum, so the total fractional error in the *p*-value (Equation 1) will be no more than ε.

GONOME saves additional time by only computing Equation 1 if the *Z*-score of the observed score, *S*, is greater than a user defined cutoff. The *Z*-score is computed using the mean and standard deviation of *S*, which can be computed efficiently. Details of the derivation of the mean and standard deviation of the correlation scoring function, including some extensions, and an analysis showing no significant expectations are lost for a cutoff of three standard deviations, are available at [13].

### Extraction of upstream motifs associated to functional groups

Isolation of over-represented upstream motifs is done by extracting all *k*-mers in the *S. cerevisiae *genome of lengths 5 to 11 that appear five or more times on either strand. For each *k*-mer, we use GONOME to determine which (if any) GO terms are correlated with its occurrences in the genome. (We restrict the analysis to upstream positions with a 2000 bp cutoff.) Each *k*-mer is labeled with the GO term with the lowest *E*-value, provided that the *E*-value is less than 0.05. The *k*-mers are then grouped by their GO term labels. Each such group represents a putative motif. The resultant motifs are then filtered to remove those with fewer than four occurrences (of any of the *k*-mers in the set) in upstream regions and those with more than two hundred occurrences. Motifs with fewer than four occurrences have little statistical support. Motifs with more than two hundred occurrences tend to be such things as TATA motifs, and we chose to ignore them as well.

We then use the MEME [25] algorithm to refine the motifs. Each set of *k*-mers is input to MEME. MEME aligns the *k*-mers and creates a position specific scoring matrix (PSSM) for the refined motif. We chose not to use the PSSM, but instead use the consensus sequence that MEME also outputed as the "final" motif. We then validate each consensus sequence determining all positions in the genome that match the consensus exactly. These positions are treated as the final occurrences of the motif and input to GONOME to see if the original GO term labeling the motif is significantly over-represented. The significant consensus sequences are then compared to known transcription factor consensus motifs.

### Datasets

The feature positions used for mouse, human, *D. melanogaster *and *A. thaliana *were extracted from their Genbank genomes. The human genome used herein was NCBI build 35, 26 Aug. 2004, the murine genome was NCBI build 33, 2 Sep. 2004, fly was the 13 Apr. 2005 version, and cress the 19 Feb. 2004 version [26]. The *S. cerevisiae *feature positions were derived from the SGD annotations, 7 Dec. 2004 [27]. GO annotations for human, mouse and *S. cerevisiae *were derived from the 200411 version of the main GO database, (5 Nov. 2004). Feature positions (Chromosome contigs, 4 Jul. 2005) and GO annotation table (15 Aug. 2005) for *S. pombe *came from the Sanger center [28]. *C. elegans *feature positions were derived from the WS147 build GFF file (22 Aug 2005) and GO table revision 1.52 [29]. *D. melanogaster *GO annotations came from the revision 1.65 of the FLYBASE GOA table [30]. *A. thaliana *GO annotations were derived from the TAIR GOA table, revision 1.821 [31]. All datasets are archived at the website [13].

CpG island data was generated using the UCSC version of Larsen's CpG island scanner [11] using default parameters, and taking the position of the 3' end on each strand. The scanner was run over the unmasked human genome, and then those positions matching regions annotated as repetitive by A. F. A. Smit and P. Green's RepeatMasker were removed. The PACE UAS sequences were extracted using S. Weng's PatMatch program at the SGD website [32]. The parameters were set to extract only exact matches.

Transcription factor consensus motifs were drawn from the SGD verified list [33], which was primarily derived from [34].

## Availability and requirements

**Project name**: GONOME

**Project homepage**: .

**Operating systems**: platform independent

**Programming languages**: Perl, C++

**Other requirements**: Optionally Connection to a GO database

**License**: open source under MIT license

**Any restrictions to use by non-academics**: No

The downloadable GONOME package includes a BIOPERL [35] based parser for extracting necessary data from Genbank or EMBL files. Input to GONOME consists of a table of the starts, ends, strand and feature IDs of genes, and a list of genomic positions. Output from GONOME is a graph giving the *E*-values of the most over-represented GO terms, and tables providing *E*-values.

The web version of GONOME [6] allows on-line querying against the human, mouse, *S. cerevisiae*, *S. pombe*, *C. elegans*, *D. melanogaster*, and *A. thaliana *(more genomes coming soon) using a set of user-provided genomic positions.

## List of abbreviations

EMBL European Molecular Biology Laboratory

GFF General Feature Format

GO Gene Ontology

NCBI National Center for Biotechnology Information

PACE Proteosome Associated Control Element

PSSM Position Specific Score Matrix

UAS Upstream Activating Sequence

UCSC University of California, Santa Cruz.

## Authors' contributions

SMS: Conceived and developed the application, implemented the analyses and drafted the manuscript.

TLB: Proposed much of the statistical methodology, advised on usage of MEME application and assisted in drafting the manuscript.

JSM: Advised, participated in interface design and assisted in drafting the manuscript.

All authors read and approved the final manuscript.
